# Multi-Task Deep Learning on MRI for Tumor Segmentation and Treatment Response Prediction in an Experimental Model of Hepatocellular Carcinoma

**DOI:** 10.3390/diagnostics15222844

**Published:** 2025-11-10

**Authors:** Guangbo Yu, Zigeng Zhang, Aydin Eresen, Qiaoming Hou, Vahid Yaghmai, Zhuoli Zhang

**Affiliations:** 1 Department of Biomedical Engineering, University of California Irvine, Irvine, CA 92697, USA; guangboy@uci.edu; 2 Department of Radiological Sciences, University of California Irvine, Irvine, CA 92868, USA; zigengz@hs.uci.edu (Z.Z.); aeresen@hs.uci.edu (A.E.); qiaominh@hs.uci.edu (Q.H.); vyaghmai@hs.uci.edu (V.Y.); 3 Chao Family Comprehensive Cancer Center, University of California Irvine, Irvine, CA 92868, USA; 4 Department of Pathology and Laboratory Medicine, University of California Irvine, Irvine, CA 92697, USA

**Keywords:** hepatocellular carcinoma, magnetic resonance imaging, multi-task deep learning, treatment response prediction, histology validation

## Abstract

**Background**: Assessing the efficacy of combination therapies in hepatocellular carcinoma (HCC) requires both accurate tumor delineation and biologically validated prediction of therapeutic response. Conventional MRI-based criteria, which rely primarily on tumor size, often fail to capture treatment efficacy due to tumor heterogeneity and pseudo-progression. This study aimed to develop and biologically validate a multi-task deep learning model that simultaneously segments HCC tumors and predicts treatment outcomes using clinically relevant multi-parametric MRI in a preclinical rat model. **Methods**: Orthotopic HCC tumors were induced in rats assigned to Control, Sorafenib, NK cell immunotherapy, and combination treatment groups. Multi-parametric MRI (T1w, T2w, and contrast enhanced MRI) scans were performed weekly. We developed a U-Net++ architecture incorporating a pre-trained EfficientNet-B0 encoder, enabling simultaneous segmentation and classification tasks. Model performance was evaluated through Dice coefficients and area under the receiver operator characteristic curve (AUROC) scores, and histological validation (H&E for viability, TUNEL for apoptosis) assessed biological correlations using linear regression analysis. **Results**: The multi-task model achieved precise tumor segmentation (Dice coefficient = 0.92, intersection over union (IoU) = 0.86) and reliably predicted therapeutic outcomes (AUROC = 0.97, accuracy = 85.0%). MRI-derived deep learning biomarkers correlated strongly with histological markers of tumor viability and apoptosis (root mean squared error (RMSE): viability = 0.1069, apoptosis = 0.013), demonstrating that the model captures biologically relevant imaging features associated with treatment-induced histological changes. **Conclusions**: This multi-task deep learning framework, validated against histology, demonstrates the feasibility of leveraging widely available clinical MRI sequences for non-invasive monitoring of therapeutic response in HCC. By linking imaging features with underlying tumor biology, the model highlights a translational pathway toward more clinically applicable strategies for evaluating treatment efficacy.

## 1. Introduction

Liver cancer remains a major global health challenge, ranking as the third leading cause of cancer death worldwide, with hepatocellular carcinoma (HCC) representing the majority of cases. Despite advancements, the 5-year survival rate in the United States remains around 21% [1]. Standard treatments, including surgical resection and liver transplantation, are often limited by poor efficacy in late-stage HCC and a high recurrence rate post-surgery [2], underscoring the need for innovative treatments and precise, non-invasive monitoring methods.

Sorafenib, an FDA-approved tyrosine kinase inhibitor, targets tumor angiogenesis and proliferation via inhibition of vascular endothelial growth factor receptors (VEGFR) and RAF [3]. Natural killer (NK) cell immunotherapy has recently emerged as a promising treatment due to its selective ability to destroy cancer cells [4]. Combining Sorafenib with NK cell therapy has demonstrated synergistic potential to enhance therapeutic outcomes [5,6,7]. However, accurately monitoring immunotherapy efficacy remains challenging. Conventional MRI-based response criteria (RECIST, mRECIST, imRECIST) predominantly assess tumor size changes and often misinterpret pseudo-progression, a common phenomenon in immunotherapy characterized by transient increases in lesion size or the emergence of new lesions [8,9,10,11].

Deep learning models, especially convolutional neural networks (CNNs), offer the ability to capture complex texture patterns in MRI images that are beyond human visual capacity [12,13]. However, CNN-based architectures such as U-Net [14], typically require large datasets, presenting significant challenges in animal studies where data scarcity and manual annotation are common limitations. Transfer learning, where a model pre-trained on large datasets is fine-tuned for specific tasks, has emerged as an effective method to overcome data scarcity and improve performance in specialized domains [15,16].

To address these challenges, we developed a multi-task deep learning model using a pre-trained EfficientNet-B0 backbone within a U-Net++ framework [17,18]. Our model simultaneously performs tumor segmentation and multi-class treatment outcome prediction, providing a universal architecture adaptable across different datasets. Moreover, to enhance the biological relevance and interpretability of our model, we incorporated histological validation by correlating MRI-derived biomarkers directly with tumor microenvironment features, including viability and apoptosis.

The study aimed to develop and validate a multi-task deep learning-based model for automatic tumor segmentation and multi-class treatment outcome prediction in HCC using conventional MRI data while correlating imaging biomarkers with histological data to provide biological validation. For clarity, Table 1 summarizes key terms used throughout this study.

## 2. Materials and Methods

**Figure 1:**
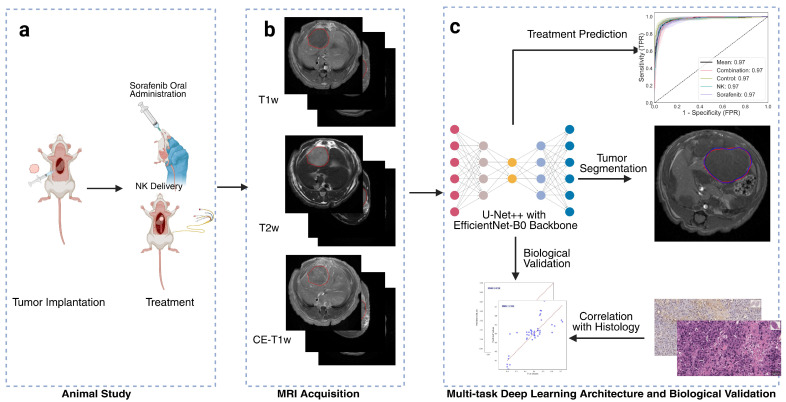
Overview of study workflow. (**a**) Animal treatment involving orthotopic tumor implantation and administration of natural killer (NK) cell immunotherapy and oral Sorafenib. (**b**) Multi-parametric magnetic resonance imaging (MRI), including T1-weighted (T1w), T2-weighted (T2w), and contrast-enhanced T1-weighted (CE-T1w) sequences, with red circles indicating the tumor regions. (**c**) Artificial intelligence (AI) model based on a multi-task U-Net++ architecture with EfficientNet-B0 encoder for simultaneous tumor segmentation and treatment outcome prediction. Deep learning biomarkers were biologically validated through correlation with histological findings.

All procedures complied with the animal protocol approved by our institution’s Institutional Animal Care and Use Committee (IACUC). The overall study design is illustrated in Figure 1. Code is available at: https://github.com/guangboyu/multi_task_HCC_classification (accessed on 6 November 2025).

### 2.1. Cell Lines and Cell Culture

Hepatoma cell lines N1-S1 (CRL-1604, ATCC, Manassas, VA, USA) for Sprague Dawley rats and McA-RH7777 (CRL-1601, ATCC, Manassas, VA, USA) for Buffalo rats were cultured in Iscove’s Modified Dulbecco’s Medium (IMDM) supplemented with 10% fetal bovine serum (FBS), 1.25% GlutaMAX (Gibco, Waltham, MA, USA), and 1% penicillin-streptomycin (Gibco, Waltham, MA, USA). The cells were maintained at 37 °C in an atmosphere of 5% CO_2_ and 95% air, subcultured every three days, and monitored for viability (>90%) using a Countess II automated cell counter (Life Technologies, Carlsbad, CA, USA) with 0.4% trypan blue dye.

RNK-16 rat NK cells, kindly provided by Thomas L. Olson (University of Virginia, Charlottesville, VA, USA), were cultured in RPMI supplemented with 25 mM 2-mercaptoethanol. Prior to therapeutic administration, RNK-16 cells were pre-treated with recombinant mouse IL-12 and IL-18 for 24 h to enhance cytotoxicity and proliferation, washed thoroughly with PBS, and rested in fresh medium for another 24 h. These pre-treated NK cells (pNK cells) were used in subsequent therapeutic interventions for both rat models.

### 2.2. Orthotopic Tumor Development

Animal experiments were conducted in compliance with protocols approved by the Institutional Animal Care and Use Committee. Sprague Dawley (SD) rats (Charles River Laboratories, Hollister, CA, USA) and Buffalo rats, bred in our animal facility, were both used in this study. Each SD rat (*n* = 24), weighing 250–300 g and aged 6–8 weeks, received subcapsular injections of 1 × 10^6^ N1-S1 cells into the left lateral lobe of the liver under 2% isoflurane anesthesia. Similarly, Buffalo rats (*n* = 24) underwent subcapsular liver injections of 2 × 10^6^ McA-RH7777 cells in 0.1 mL of PBS after a laparotomy incision exposed to the liver. For both groups, surgical regions were shaved, cleaned with iodine, and the liver tissue was temporarily fixed to prevent movement-related leakages during tumor implantation. Hemostatic gauze was applied post-injection to control bleeding, and the liver was carefully repositioned before the incision was closed using a two-layer suture technique.

### 2.3. Therapeutic Strategies: NK Cell Immunotherapy and Sorafenib Administration

Once tumors reached approximately 5 mm in size, as determined by MRI, SD, and Buffalo rats were randomly divided into four groups: NK cell immunotherapy, sorafenib treatment, combined NK cell and sorafenib treatment, and a control group (n = 6 per group for both SD and Buffalo rats). Sorafenib was administered daily via gastric gavage at a dose of 10 mg/kg for seven days. In the NK cell and combination treatment groups, both SD and Buffalo rats received 1 × 10^7^ pNK cells in 0.5 mL of PBS. For SD rats, NK cells were delivered via intrahepatic arterial (IHA) catheter, while Buffalo rats received NK cells through tail vein injection, followed by a saline flush. Control and sorafenib-only groups received PBS on the same schedule. All procedures were conducted in accordance with institutional ethical standards, and animals were monitored post-treatment for any signs of discomfort.

### 2.4. MRI Acquisition

MRI scans started one week after tumor implantation using a 3T MRI scanner (Philips Achieva, Best, The Netherlands) with a commercial wrist coil. Anesthesia was administered with 1–2% isoflurane at 2 L/min. When tumors reached 5 mm in diameter, a baseline MRI was obtained, followed by weekly scans. Buffalo rats were scanned for up to four weeks, while Sprague Dawley (SD) rats were scanned for up to three weeks.

The MRI sequences included T1-weighted (T1w), T2-weighted (T2w), and contrast-enhanced T1-weighted (CE-T1w) sequences after Gadolinium administration. Specific MRI sequence protocols were as follows: (a) Axial T2-weighted MRI with a repetition time (TR) of 3500 ms, echo time (TE) of 63.177 ms, slice thickness (ST) of 2 mm (without gap), flip angle (FA) of 90°, field of view (FOV) of 50 × 50 mm^2^, and number of signals averaged (NSA) of 4. (b) T1-weighted fast field echo (FFE) before and after Gadolinium contrast tail vein injection with TR of 200 ms, TE of 2.45 ms, ST of 2 mm (without gap), FA of 90°, FOV of 50 × 50 mm^2^, and NSA of 4.

Tumor regions were delineated on T1w, T2w, and CE-T1w images using ITK-SNAP software (v.4.2.0). Affine transformation was applied to maintain spatial consistency across sequences. Tumor segmentation was independently performed by three radiologists with 2, 5, and >20 years of experience in liver MRI. Discrepancies between the two junior readers were reviewed and finalized by the senior radiologist. Inter-rater reproducibility, evaluated from eight randomly selected cases, yielded a mean Dice coefficient of 0.928 ± 0.053, confirming high annotation consistency.

### 2.5. Model Architecture

**Figure 2:**
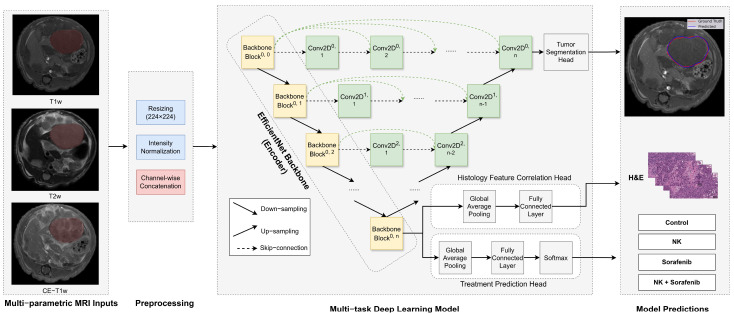
Architecture of the multi-task deep learning model. The U−Net++ model incorporates an EfficientNet−B0 encoder to extract hierarchical imaging features from MRI slices (yellow blocks). The decoder (green blocks) restores spatial resolution using nested skip connections (dashed arrows) to generate tumor segmentation masks. A separate classification head processes encoded features to predict treatment outcomes. Arrows indicate the direction of data flow.

The U-Net++ architecture was employed for tumor segmentation, with an additional classification head for predicting treatment outcomes (Figure 2). This architecture features an encoder–decoder structure with nested skip connections to capture fine-grained details and improve segmentation accuracy. The encoder, based on the pre-trained EfficientNet-B0, leverages transfer learning to address the limited availability of annotated medical data and has flexible depth, allowing substitution with more advanced encoders if needed. The decoder mirrors the encoder with transposed convolutions for upsampling, while skip connections enable effective feature reuse. To incorporate temporal information from the longitudinal study design, a 512-dimensional learnable time embedding was used to represent the imaging week for each subject. This embedding was concatenated with the encoded feature representation before classification to provide temporal context and allow the model to adjust for treatment dynamics over time. For treatment outcome classification, a fully connected head with ReLU activations, dropout (0.5), average pooling, and a Softmax layer provided probabilities for the four treatment groups: Control, NK, Sorafenib, and NK + Sorafenib.

### 2.6. Training Procedure

MRI images were preprocessed (Supplementary Material File S1) by normalizing pixel intensities to reduce variability, then resized to 224 × 224 pixels to match the input size of EfficientNet-B0. Data augmentation techniques, including rotation, flipping, and zooming, were applied to improve model robustness.

The training procedure involved a weighted loss function combining Dice Loss for segmentation and Cross-Entropy Loss for classification: $L=\alpha \cdot L_{seg}+\left(1-\alpha \right)\cdot L_{cls}$, where L represents the total loss, $L_{seg}$ represents the segmentation loss, $L_{cls}$ represents the classification loss, and α is a weight coefficient optimized from 0.1 to 0.9 in increments of 0.05, with the final α set to 0.75. The Adam optimizer was used with an initial learning rate of $5\times 10^{-4}$. A batch size of 32 was used for training, and the model was trained for 30 epochs with early stopping based on the validation loss to prevent overfitting. To enhance generalizability, five-fold cross-validation was performed at the animal level using the *GroupKFold* method in scikit-learn [19], grouping slices by animal ID. This ensured that all MRI slices from the same animal across all timepoints were contained within a single fold, thereby preventing potential data leakage and preserving independence between training and validation sets.

### 2.7. Radiomics Analysis

To provide a comparative baseline, radiomic features were extracted from the multi-parametric MRI sequences using Pyradiomics (version 3.1.0, PyRadiomics Community) [20]. Among the features available, entropy and contrast were selected because they are widely used to characterize tumor heterogeneity and local intensity variation in oncologic imaging. These handcrafted features were analyzed independently and were not incorporated into the deep learning framework. This parallel analysis enabled a direct comparison between the limited discriminative capacity of conventional radiomics metrics and the more complex representations automatically learned by the multi-task deep learning model.

### 2.8. Histological Analysis

At the end of the study, animals were euthanized in accordance with IACUC guidelines. Tumor-bearing livers were excised, fixed in 10% formalin, paraffin-embedded, and sectioned into 5 µm-thick slices. Histological assessments included hematoxylin and eosin (H&E) staining for tumor viability and TUNEL staining to detect apoptosis.

Slides were digitized using a Hamamatsu whole slide scanner and analyzed with QuPath (v0.4.3) and ImageJ (v1.54c). Tumor viability was quantified by calculating the percentage of viable tumor cells from five randomly selected H&E-stained sections at 100× magnification. Apoptosis was quantified by TUNEL staining, measuring the rate of apoptotic cells in five randomly selected fields at 100× magnification. All quantifications were conducted by blinded researchers.

### 2.9. Statistical Analysis

Radiomic features (entropy and contrast) extracted using Pyradiomics [20] were statistically analyzed across treatment groups (Control, Sorafenib, NK, NK + Sorafenib) and timepoints (Week 1, 2, 3). Statistical significance was assessed using two-way ANOVA and T-tests in GraphPad Prism 10, with thresholds set at * *p* < 0.05; ** *p* < 0.01; *** *p* < 0.001; **** *p* < 0.0001.

The multi-task deep learning model’s performance was assessed using Dice Coefficient, Intersection over Union (IoU), Precision, and Recall for segmentation, and Accuracy, Precision, Recall, and Area Under the Receiver Operating Characteristic Curve (AUROC) with 95% CI for classification, averaged over 5-fold cross-validation.

To assess the correlation between MRI-derived deep learning features and histological biomarkers (tumor viability and apoptosis), a linear regression model was used. After training the multi-task model for treatment outcome prediction, the encoder parameters were frozen, and the 1280-dimensional feature vectors extracted from the final encoder layer after global average pooling were used as inputs. A regression head consisting of adaptive average pooling, two fully connected layers with ReLU activation and dropout of 0.5, and a final linear layer was trained to predict histological metrics based on H&E and TUNEL staining. Histology measurements were obtained at the terminal timepoint immediately after the final MRI scan to ensure temporal and biological correspondence between imaging and histology. Model performance was evaluated using Root Mean Square Error (RMSE) to quantify the relationship between MRI features and histological data.

## 3. Results

### 3.1. Dataset and Radiomics Analysis of MRI Sequences

**Figure 3:**
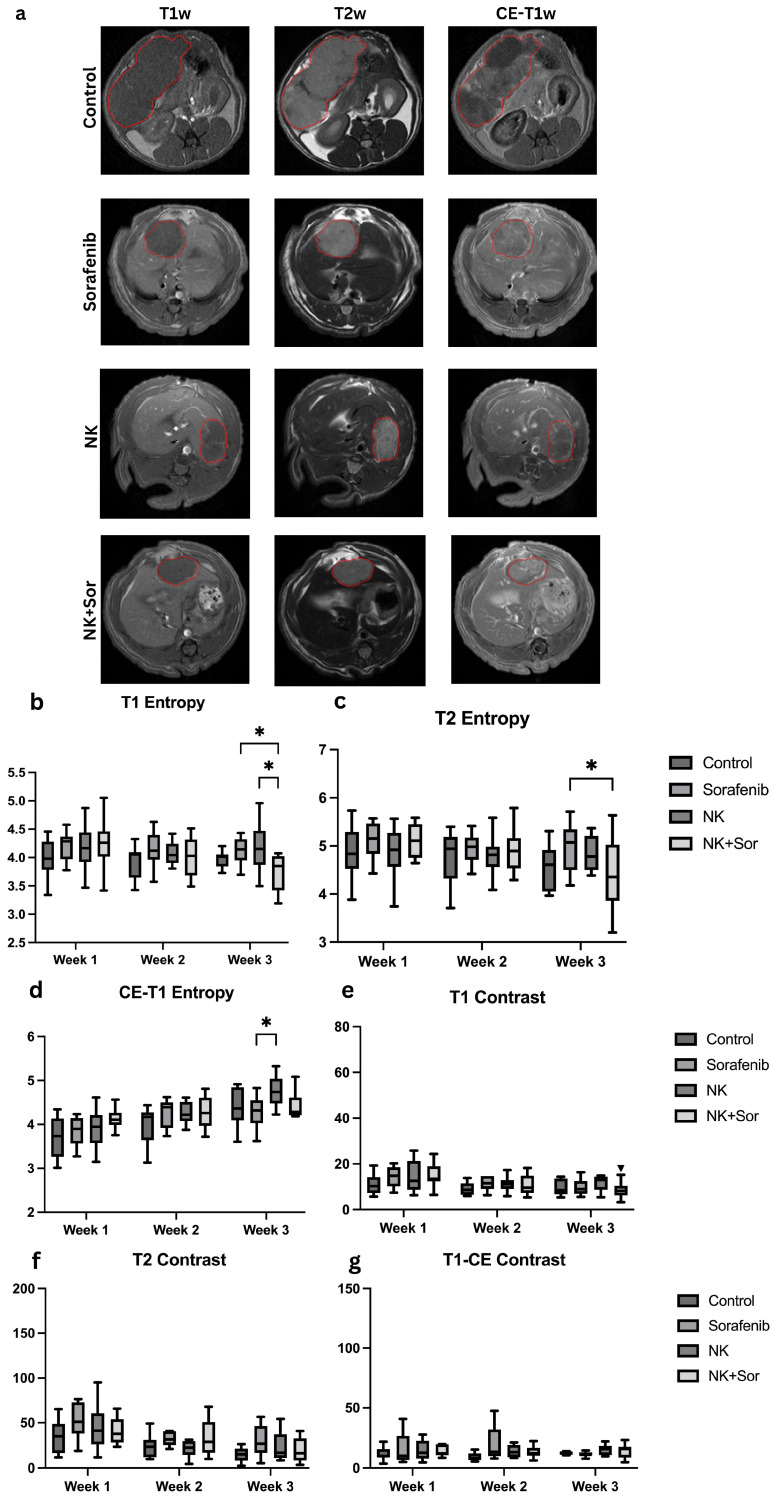
Representative MRI images and radiomics features analysis. (**a**) Representative axial MRI scans (T1w, T2w, CE-T1w) at treatment week 3 for each group: control, Sorafenib, NK cells, and combination therapy. Tumors are outlined in red to indicate regions of interest. (**b**–**d**) Entropy and (**e**–**g**) contrast values computed from T1w, T2w, and CE-T1w sequences, respectively, across treatment groups. Asterisks indicate statistically significant differences (*: *p* < 0.05).

A total of 2175 MRI slices were acquired across four treatment groups: Control, Sorafenib, NK cell immunotherapy, and NK cell immunotherapy combined with Sorafenib. Each group consisted of 12 rats (*n* = 48 total). Every rat underwent multi-parametric MRI at four timepoints, including T1-weighted (T1w), T2-weighted (T2w), and contrast-enhanced T1-weighted (CE-T1w) sequences. Each sequence contained approximately 20 slices, adjusted as needed to cover the tumor volume fully. Slices containing visible tumor regions were manually reviewed and included to ensure balanced sampling across animals and timepoints. These three MRI sequences were combined as a three-channel input to capture complementary information for more comprehensive feature extraction. Representative MRI images are shown in Figure 3a.

Radiomics analysis was performed to evaluate textural features that may reflect treatment-induced tumor changes. Specifically, entropy and contrast were extracted, representing pixel intensity randomness and local intensity variation, respectively. While statistically significant differences in these features were observed between certain treatment groups (Figure 3b–g), the ability of traditional radiomics metrics to reliably discriminate treatment response remained limited. These findings underscore the need for more advanced modeling approaches to extract informative biomarkers and accurately assess therapeutic efficacy in hepatocellular carcinoma.

### 3.2. Tumor Segmentation Performance

**Figure 4:**
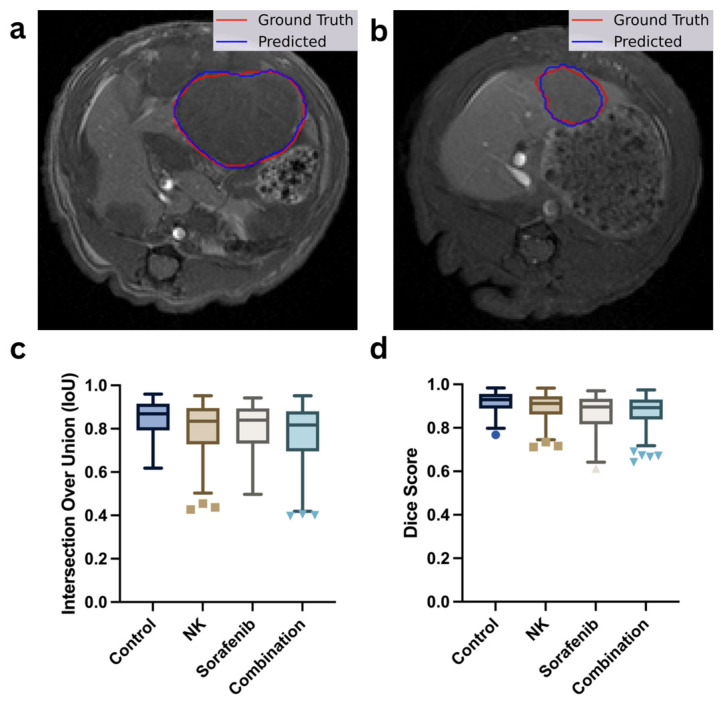
Evaluation of tumor segmentation performance. (**a**,**b**) Representative T1-weighted MRI scans showing manual tumor outlines (red) versus AI-predicted tumor masks (blue). (**c**,**d**) Box plots comparing segmentation accuracy using Intersection-over-Union (IoU) and Dice similarity coefficient scores across the control, Sorafenib, NK cells, and combination therapy groups. Different marker shapes correspond to individual data points from each group (e.g., circles for Control, squares for NK, triangles for Sorafenib, and inverted triangles for Combination).

The segmentation performance of the multi-task U-Net++ model is depicted in Figure 4, where the model accurately delineated tumor boundaries from T2-weighted MRI images. The model achieved a mean Precision of 0.88, Recall of 0.86, Dice coefficient of 0.92, and an Intersection over Union (IoU) score of 0.86 across all validation folds, indicating a substantial overlap between predicted and actual tumor regions.

Figure 4a,b show the representative segmentation results, illustrating the close alignment between the actual tumor boundaries (red outlines) and the predicted segmentation masks (blue outlines). Figure 4c,d present the model’s performance metrics across the four treatment groups (Control, NK, Sorafenib, and Combination), quantified by IoU and Dice scores, respectively. The results demonstrate consistent segmentation performance across all treatment groups, demonstrating the model’s unbiased performance across different treatment groups.

### 3.3. Classification of Multi-Class Treatment Outcomes

The multi-task U-Net++ model with an EfficientNet-B0 encoder, was evaluated for its ability to classify treatment outcomes across four groups: Control, Sorafenib, NK cell therapy, and Sorafenib + NK combination therapy. To account for the limited sample size and enhance generalizability, 5-fold cross-validation was employed. The model achieved an average accuracy of 84.97% (95% CI: 78.76–90.21%), sensitivity of 85.34% (95% CI: 79.20–90.75%), and specificity of 85.52% (95% CI: 79.50–90.77%).

**Figure 5:**
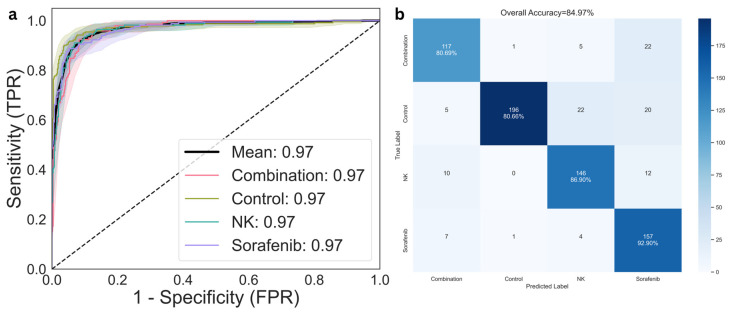
Classification performance for treatment response prediction. (**a**) Receiver operating characteristic (ROC) curves for multi-class classification across the control, NK cells, Sorafenib, and combination therapy groups. (**b**) Confusion matrix comparing predicted and actual treatment groups, illustrating model accuracy and error distribution.

Receiver operating characteristic (ROC) analysis was conducted using a one-vs-rest approach to assess discriminative performance across classes (Figure 5a). The area under the curve (AUROC) was 0.97 for each of the four treatment groups—Control, Sorafenib, NK, and combination therapy—resulting in a mean AUROC of 0.974 (95% CI: 0.956–0.987). The confusion matrix (Figure 5b) presents the distribution of predicted versus actual labels across all groups, indicating relatively low misclassification rates and consistent performance across validation folds. To further evaluate class-level discrimination, per-class precision, recall, and F1-scores were computed (Table 2). The model demonstrated balanced classification across all treatment groups, with macro-averaged precision, recall, and F1-scores of 0.85, 0.85, and 0.84, respectively.

### 3.4. Correlation of MRI-Derived Features with Histological Biomarkers

Following the demonstration of model accuracy in predicting treatment outcomes, we next investigated the biological basis underlying these predictions. To explore why the model could reliably distinguish between treatment groups, we analyzed the relationship between MRI-derived deep learning features and gold-standard histological markers of tumor viability and apoptosis.

**Figure 6:**
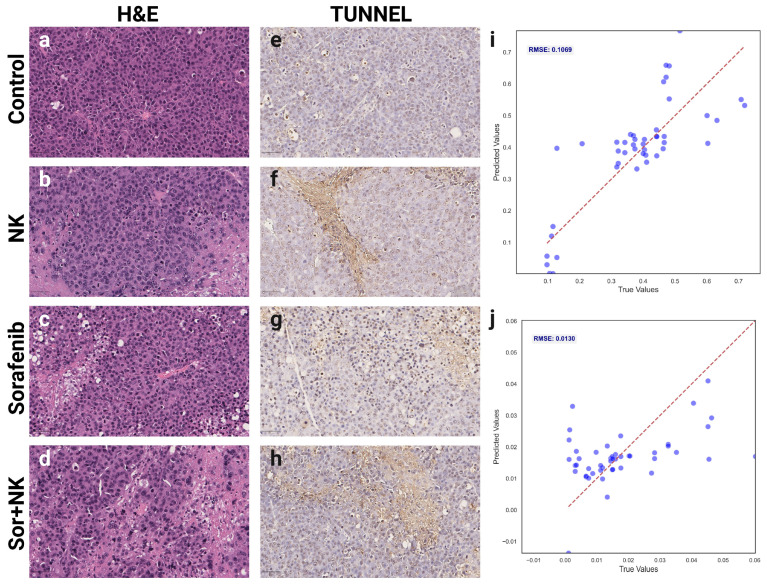
Histological validation of MRI-derived deep learning biomarkers. (**a**–**d**) Representative hematoxylin and eosin (H&E)-stained histological images demonstrating tumor morphology and cell viability across groups: (**a**) control, (**b**) NK cells, (**c**) Sorafenib, (**d**) combination therapy. (**e**–**h**) Terminal deoxynucleotidyl transferase dUTP nick end labeling (TUNEL)-stained histological sections indicating apoptosis levels for each group: (**e**) control, (**f**) NK cells, (**g**) Sorafenib, (**h**) combination therapy. (**i**,**j**) Scatter plots demonstrating correlations between MRI-derived deep learning biomarkers and histological measurements of (**i**) tumor viability from H&E staining and (**j**) apoptosis index from TUNEL staining.

Histological evaluations assessed tumor viability and apoptosis across the four treatment groups (Control, Sorafenib, NK, and Sorafenib + NK). H&E staining (Figure 6a–d) showed reduced viable tumor cells in the treatment groups compared to the Control, while TUNEL staining (Figure 6e–h) indicated higher apoptosis levels across all treatments. Root Mean Square Error (RMSE) values were calculated to quantify the correlation between MRI-derived deep learning features and histological biomarkers, with RMSEs of 0.1069 for H&E (tumor viability; Figure 6i) and 0.013 for TUNEL (apoptosis; Figure 6j), indicating strong agreement between predicted and observed histological outcomes.

## 4. Discussion

Improving the accuracy and biological relevance of MRI-based treatment monitoring for HCC remains a critical challenge. In this study, we developed a multi-task deep learning model that simultaneously performs tumor segmentation and treatment outcome prediction, utilizing a pre-trained EfficientNet-B0 encoder within a U-Net++ framework. This design enhances both segmentation accuracy and classification efficiency, achieving an AUROC of 0.97 and an IoU of 0.86. The combination of T1w, T2w, and CE-T1w MRI texture features provided effective non-invasive biomarkers for evaluating treatment responses in HCC rat models treated with NK cells, sorafenib, and their combination. Correlating MRI-derived deep learning biomarkers with histological data ensured the biological validation of our imaging results.

Traditional criteria for treatment monitoring, such as iRECIST, mRECIST, and imRECIST [9,10,11], primarily rely on tumor size changes and often fail to capture immunotherapy responses due to tumor heterogeneity or pseudoprogression [8]. Recent studies have explored AI to improve imaging-based cancer diagnosis and treatment assessment [21]. Radiomics studies have demonstrated that quantitative MRI features can reflect treatment-induced microstructural changes and correlate with histological biomarkers of response [22]. However, such approaches depend on handcrafted feature extraction and are limited by challenges of standardization and reproducibility, particularly when applied across heterogeneous datasets. More recently, transformer-based models have been used to analyze ROI-level MRI regions for outcome prediction [23], yet they capture a more limited spatial context than whole-image learning frameworks. In contrast, deep learning models offer better generalization but typically require large datasets. For example, von Schacky et al. [24] introduced a multi-task deep learning model that performed bounding box placement, segmentation, and classification of primary bone tumors on radiographs. This streamlined approach improved efficiency and matched the accuracy of fellowship-trained radiologists, though it required extensive data. Similarly, Li et al. [25] applied a multi-task deep learning model to HCC patients for response prediction and tumor segmentation after trans-arterial chemoembolization (TACE). Although these models performed well, the absence of histological validation highlighted the need for biologically grounded biomarkers.

Our approach addresses limitations by employing a multi-task framework with a pre-trained EfficientNet-B0 encoder within the U-Net++ architecture, enhancing accuracy and robustness in tumor segmentation and treatment classification while reducing the need for large datasets. Integrating multi-parametric MRI (mpMRI) sequences—including T1w, T2w, and CE-T1w images—captures intricate texture details, thereby improving segmentation precision and outcome prediction. This multi-task design streamlines MRI image analysis, reducing manual annotation and enabling efficient assessment of treatment responses. Finally, correlating MRI-derived deep learning biomarkers with gold-standard histology bridges the gap between preclinical and clinical studies, providing biological validation.

Several limitations should be noted. The dataset was limited to MRI images from an HCC rat model, potentially impacting generalizability to human clinical data. Differences in tumor biology, immune microenvironment, and MRI acquisition parameters between species may affect translational applicability. Nevertheless, the framework established here provides a foundation for future work evaluating cross-species adaptation and validating MRI-derived deep learning biomarkers in clinical datasets. Recent work [26] further supports the feasibility of transfer learning from preclinical to clinical domains when biologically conserved representations and harmonized imaging protocols are leveraged, underscoring the translational potential of our approach. All imaging was performed on a clinical-grade 3 T MRI system, and the pre-trained EfficientNet-B0 encoder further enhances translational relevance by leveraging domain-invariant representations learned from large-scale imaging datasets. Additionally, relying exclusively on MRI without complementary biomarkers, such as α-Fetoprotein (AFP) [27], may reduce comprehensive tumor characterization and impact the accuracy of outcome predictions. Third, the study primarily assessed early therapeutic responses, limiting the evaluation of long-term efficacy and the model’s ability to detect late-onset responses or recurrences.

Future studies should address these limitations by using diverse datasets, integrating multi-omics biomarkers, and conducting longitudinal analyses for improved robustness and clinical applicability. In parallel, the development of a composite prognostic signature—integrating multiple histological endpoints rather than relying on individual markers—may enhance the robustness and translational relevance of imaging-based predictions. Such a biologically informed signature could enable more reliable stratification of treatment response and serve as a foundation for clinical risk models in hepatocellular carcinoma.

## 5. Conclusions

This study presents a biologically validated multi-task deep learning framework for non-invasive monitoring of therapeutic response in HCC using multi-parametric MRI. By correlating imaging-derived features with histological markers of tumor viability and apoptosis, the model demonstrates biological specificity and mechanistic relevance. These findings suggest that deep learning–based imaging biomarkers can improve response assessment and contribute to the development of personalized treatment strategies for HCC and potentially other solid cancers.

## Figures and Tables

**Table 1:** Summary of key technical terms used in this study.

Term	Description & Relevance in This Study
RECIST	Response Evaluation Criteria in Solid Tumors. A standardized guideline that quantifies tumor response to therapy based on measurable size changes. Mentioned to highlight traditional limitations of size-based criteria in evaluating therapeutic response.
mRECIST	Modified RECIST. Incorporates measurements of the viable enhancing portion of tumors after therapy. Discussed as an advancement over RECIST but still limited for immunotherapy evaluation.
imRECIST	*Immune-modified RECIST.* Adjusts RECIST criteria to account for immune-related responses such as pseudoprogression.
U-Net++ architecture	A convolutional encoder–decoder network with nested skip connections that improve segmentation accuracy. Used as the backbone for precise tumor delineation.
EfficientNet-B0 encoder	A pre-trained convolutional encoder optimized for efficiency and accuracy. Used here to extract multi-scale features from MRI data and improve generalization with limited samples.
Convolutional Neural Network (CNN)	A deep-learning model that captures spatial features in imaging data. Forms the fundamental structure underlying both the encoder and decoder parts of the network.
Multi-task deep learning model	A neural-network framework designed to perform multiple related tasks simultaneously—in this study, tumor segmentation and treatment outcome classification—to enhance efficiency and performance.
Transfer learning	Leveraging pre-trained weights from large-scale datasets to improve model performance on smaller medical datasets.
Dice coefficient	Statistical metric quantifying overlap between predicted and ground-truth segmentation masks. Used to assess segmentation accuracy.
AUROC (Area Under the Receiver Operating Characteristic Curve)	Measures model discrimination ability across classes. Applied to evaluate classification performance for treatment prediction.

**Table 2:** Per-class classification metrics derived from the confusion matrix.

Class	Precision	Recall	F1-Score
Control	0.9899	0.8066	0.8889
NK	0.8249	0.8690	0.8464
Sorafenib	0.7441	0.9290	0.8263
Combination	0.8417	0.8069	0.8239
Macro Average	0.8501	0.8529	0.8464
Weighted Average	0.8647	0.8497	0.8515

## Data Availability

The data supporting this study are available upon reasonable request to the corresponding author.

## Supplementary Materials

The following supporting information can be downloaded at: https://www.mdpi.com/article/10.3390/diagnostics15222844/s1, File S1: MRI preprocessing configuration.

## Abbreviations

The following abbreviations are used in this manuscript:

AI	Artificial Intelligence
AUROC	Area Under the Receiver Operating Characteristic
CE-T1w	Contrast-Enhanced T1-Weighted
CNN	Convolutional Neural Network
FA	Flip Angle
FOV	Field of View
HCC	Hepatocellular Carcinoma
IoU	Intersection Over Union
NK	Natural Killer
NSA	Number of Signals Acquisitions
RMSE	Root Mean Square Error
ST	Slice Thickness
TE	Echo Time
TR	Repetition Time
T1w	T1-Weighted
T2w	T2-Weighted
