# Supplementary material for: Multi-Task Deep Learning on MRI for Tumor Segmentation and Treatment Response Prediction in an Experimental Model of Hepatocellular Carcinoma
Source: Diagnostics (Basel). 2025 Nov 10;15(22):2844. doi: 10.3390/diagnostics15222844 (PMC12650808; doi:10.3390/diagnostics15222844)
Supplement: Supplementary file 1 [file diagnostics-15-02844-s001.zip › diagnostics-3944459-supplementary.pdf]

Supplementary Materials

File S1. MRI preprocessing configuration

All MRI slices were preprocessed prior to training to ensure consistent spatial dimensions and intensity distributions. Each slice was resampled to 224 × 224 pixels and normalized on a per-image basis to have zero mean and unit variance (mean = 0.0, standard deviation = 1.0, maximum pixel value = 255).

Data augmentation strategies were applied during training to enhance model robustness and prevent overfitting. Random transformations included horizontal and vertical flips, 90° rotations, affine transformations (shift limit = 0.1, scale limit = 0.1, rotation limit = 15°), and brightness–contrast adjustments (contrast limit = 0.3), each applied with a probability of 0.5.
